# Impact of *ABCB1* 1236C > T-2677G > T-3435C > T polymorphisms on the anti-proliferative activity of imatinib, nilotinib, dasatinib and ponatinib

**DOI:** 10.1038/srep29559

**Published:** 2016-07-12

**Authors:** Géraldine Dessilly, Nadtha Panin, Laure Elens, Vincent Haufroid, Jean-Baptiste Demoulin

**Affiliations:** 1 Louvain centre for Toxicology and Applied Pharmacology, Institut de Recherche Expérimentale et Clinique, Université catholique de Louvain, Brussels, Belgium; 2 Louvain Drug Research Institute, Université catholique de Louvain, Brussels, Belgium; 3 Department of Clinical Chemistry, Cliniques Universitaires Saint-Luc, Université catholique de Louvain, Brussels, Belgium; 4 De Duve Institute, Université catholique de Louvain, Brussels, Belgium

## Abstract

Overexpression of ABCB1 (also called P-glycoprotein) confers resistance to multiple anticancer drugs, including tyrosine kinase inhibitors (TKIs). Several *ABCB1* single nucleotide polymorphisms affect the transporter activity. The most common *ABCB1* variants are 1236C > T, 2677G > T, 3435C > T and have been associated with clinical response to imatinib in chronic myelogenous leukaemia (CML) in some studies. We evaluated the impact of these polymorphisms on the anti-proliferative effect and the intracellular accumulation of TKIs (imatinib, nilotinib, dasatinib and ponatinib) in transfected HEK293 and K562 cells. ABCB1 overexpression increased the resistance of cells to doxorubicin, vinblastine and TKIs. Imatinib anti-proliferative effect and accumulation were decreased to a larger extent in cells expressing the ABCB1 wild-type protein compared with the 1236T-2677T-3435T variant relatively to control cells. By contrast, *ABCB1* polymorphisms influenced the activity of nilotinib, dasatinib and ponatinib to a much lesser extent. In conclusion, our data suggest that wild-type ABCB1 exports imatinib more efficiently than the 1236T-2677T-3435T variant protein, providing a molecular basis for the reported association between *ABCB1* polymorphisms and the response to imatinib in CML. Our results also point to a weaker impact of *ABCB1* polymorphisms on the activity of nilotinib, dasatinib and ponatinib.

Tyrosine kinase inhibitors (TKIs) have been approved for the treatment of various cancers driven by kinase oncogenes such as *EGF receptors*, *ALK*, *KIT* and *BCR-ABL1*. In this study, we have focused on clinically approved TKIs targeting BCR-ABL, a fusion protein which results from the reciprocal translocation between *BCR* (breakpoint cluster region) on chromosome 22 and *ABL1* (Abelson kinase) on chromosome 9^1^. These drugs, namely, imatinib, nilotinib, dasatinib and ponatinib have spectacularly improved the survival of patients with chronic myeloid leukaemia (CML)^2^,^3^,^4^,^5^. Imatinib (STI571) was approved by the Food and Drug Administration (FDA) in 2001^6^,^7^. Nilotinib (AMN107) and dasatinib (BMS-354825) are second generation TKIs that were developed to overcome imatinib resistance or intolerance, which occurs in approximatively 20 to 30% of CML patients^8^. Acquisition of mutations within the kinase domain of ABL is a major cause of resistance to TKIs but additional mechanisms have also been incriminated including overexpression of ABC efflux protein^5^,^9^,^10^. First and second generation TKIs are ineffective against the BCR-ABL T315I “gatekeeper” mutation, which blocks the access of the drug to the ATP-binding site of the enzyme. To solve this issue, the third generation TKI ponatinib was developed.

ABC transporters use ATP to actively transport substrates, across biological membranes^11^. The overexpression of *ABCB1* confers resistance to a wide variety of chemotherapeutic substrates including vinca alkaloids (e.g. vinblastine) and anthracyclines (e.g. doxorubicin)^12^. They were also suggested to play a role in the development of resistance against TKIs (i.e. imatinib, nilotinib and dasatinib)^13^,^14^,^15^. Beside the expression level of *ABCB1*, polymorphisms may also modulate ABCB1 activity and by consequence drug efficiency. More than 60 coding single nucleotide polymorphisms (SNPs) have been reported in the *ABCB1* gene (www.pharmgkb.org)^16^,^17^,^18^,^19^. The three most common variants in the *ABCB1* coding region are rs1128503 (1236C > T, Gly412Gly), rs2032582 (2677G > T/A, Ala893Ser/Thr) and rs1045642 (3435C > T, Ile1145Ile). They present a Minor Allele Frequency (MAF) of approximatively 50% in the Caucasian population and are in linkage disequilibrium. Several clinical trials have studied the impact of these three SNPs on the clinical response to imatinib. In one study, it was shown that the 1236C-2677G-3435C wild-type haplotype is associated with a decreased rate of major molecular response to imatinib (decreased frequency from 70% to 44.6%). In the same report, homozygous patients for the allele 1236T presented the best molecular response and the highest imatinib plasma concentrations^20^. Another study also showed that the 1236C-2677G-3435C haplotype was associated with higher resistance to imatinib^21^. However, other reports, including two meta-analyses, failed to confirm the impact of this haplotype either on the molecular response or on drug resistance in patients treated with imatinib^22^,^23^. Consequently, the effect of these SNPs towards imatinib remains controversial. However, results based on population studies are sometimes indecisive mainly because of the presence of numerous uncontrolled confounding factors. As a complement to population studies, recombinant cell lines are very useful to test the functional impact of genetic variants. Along this idea, two studies have analysed the TKIs transport activity of ABCB1 in transfected cultured cells. However, they did not confirm the involvement of the 1236T-2677T-3435T variant in imatinib transport or anti-proliferative effect^24^,^25^.

To further analyse the impact of *ABCB1* polymorphisms, we set up two different cell models using HEK293 (human embryonic kidney) and K562 (human erythroleukemic) cell lines, as previously described^26^,^27^. HEK293 is a commonly used model to test ABC transporter variants whereas K562 is derived from a human myeloid leukaemia carrying the *BCR-ABL1* fusion and is therefore particularly suitable for testing the activity of TKIs. In this report, we have evaluated the influence of these SNPs on ABCB1 activity towards imatinib. We have also tested other TKIs, nilotinib, dasatinib and ponatinib, which are reported ABCB1 substrates^28^,^29^,^30^. Little information is available regarding the impact of *ABCB1* polymorphisms on these three drugs.

## Results

### Generation of *ABCB1* 1236C > T-2677G > T-3435C > T recombinant cell lines

**Figure 1:**
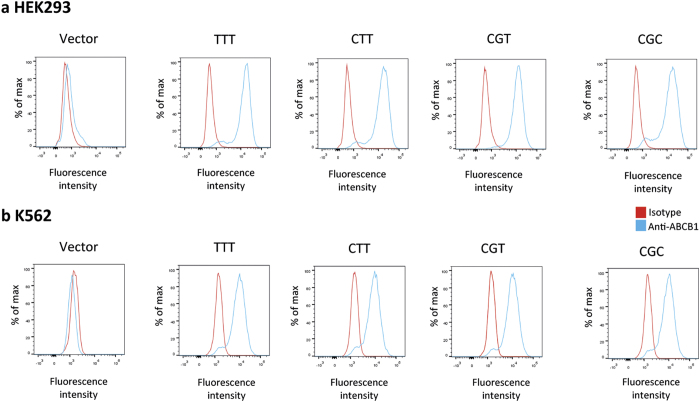
ABCB1 cell surface expression. Histograms created from a flow cytometry analysis of (**a**) HEK293 stably transfected with the empty pcDNA3.1 (vector) and HEK293 cells transfected with *ABCB1*_T-T-T_*, ABCB1*_C-T-T,_ *ABCB1*_C-G-T_ and *ABCB1*_C-G-C_ (median fluorescence intensity, arbitrary units (AI): 15384, 12381, 16290 and 16521, respectively) and (**b**) K562 cells stably transfected with the empty pEF (vector), and with K562 cells transfected with *ABCB1*_T-T-T_*, ABCB1*_C-T-T_*, ABCB1*_C-G-T_ and *ABCB1*_C-G-C_ (median fluorescence: 10271, 9577, 8993 and 9577, respectively). Cells were incubated with an FITC-conjugated anti-ABCB1 antibody (blue line) or a matched isotypic control (red line).

After transfection of HEK293 and K562 cells with pcDNA3.1 and pEF-myc-cyto vectors (*ABCB1*_C-G-C_, *ABCB1*_C-G-T_, *ABCB1*_C-T-T_ or *ABCB1*_T-T-T_) respectively, recombinant cell lines expressing *ABCB1* (thereafter called HEK _C-G-C_, HEK _C-G-T_, HEK  _C-T-T_, HEK _T-T-T_ or K562_C-G-C_, K562_C-G-T_, K562_C-T-T_, K562_T-T-T_) or cell lines transfected with the empty vector (called HEK _pcDNA3.1_ or K562_pEF_) were selected in the presence of G418. Similar ABCB1 surface expression was ensured by sorting recombinant cells by fluorescence activated cell sorting (FACS) with fluorescence parameters gated on the same level of intensity. As depicted in Fig. 1a,b, comparable surface protein expression levels were demonstrated by analytic flow cytometry in recombinant models. No fluorescence signal was detected in HEK _pcDNA3.1_ or K562_pEF_ cell lines, suggesting negligible endogenous expression.

**Figure 2:**
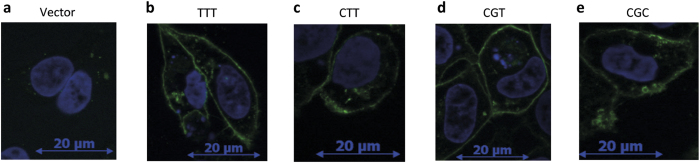
ABCB1 expression analysis by fluorescence microscopy. (**a**) HEK293 transfected with empty plasmid pcDNA3.1 (vector) and HEK293 transfected with (**b**) *ABCB1*_T-T-T_, (**c**) *ABCB1*_C-T-T_, (**d**) *ABCB1*_C-G-T_, (**e**) *ABCB1*_C-G-C_ cells were stained with anti-ABCB1 antibody (green fluorescence). DAPI was used to stain nuclei (blue).

The subcellular localization of wild-type and variant ABCB1 proteins was evaluated by immunofluorescence staining of HEK293 cell lines (Fig. 2). A circular fluorescent staining was observed in recombinant models (Fig. 2b–e) and indicated a membrane localization of ABCB1.

### Impact of *ABCB1* 1236C > T-2677G > T-3435C > T polymorphisms on the intracellular accumulation of rhodamine 123

**Figure 3:**
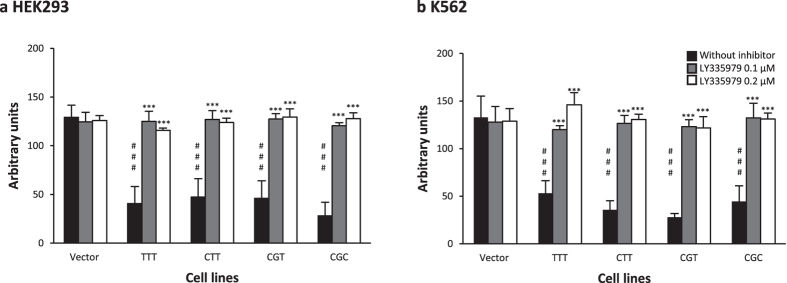
Intracellular accumulation of rhodamine 123 is not influenced by *ABCB1* 1236C > T-2677G > T-3435C > T polymorphisms. Intracellular accumulation of Rh123 (5 μM) in presence (0.1 and 0.2 μM) or absence of ABCB1 inhibitor (LY335979) (N = 6) in (**a**) HEK _pcDNA3.1_, HEK _1236C_ _>_ _T-2677G_ _>_ _T-3435C_ _>_ _T_ and (**b**) K562_pcDNA3.1_, K562_1236C_ _>_ _T-2677G_ _>_ _T-3435C_ _>_ _T_. ^#^Compared to vector ^#^p < 0.05 ^##^p < 0.01 ^###^p < 0.001, *compared without inhibitor *p < 0.05 **p < 0.01 ***p < 0.001. This experiment was performed as previously reported^26^.

We evaluated the ability of 1236C > T-2677G > T-3435C > T variants to export rhodamine (Rh) 123, a well characterized fluorescent substrate of ABCB1^26^. After incubation in the presence of Rh123, fluorescence levels were lower in all recombinant HEK293 cell lines compared to controls (Fig. 3a, p < 0.001), indicating a higher Rh123 efflux in *ABCB1* transfected cells. LY335979, a specific ABCB1 inhibitor, restored Rh123 intracellular fluorescence in transfected cell lines (Fig. 3a, p < 0.001), indicating that the differences in fluorescence intensity can be ascribed to ABCB1 expression in HEK _C-G-C_, HEK _C-G-T_, HEK _C-T-T_ and HEK_T-T-T_. There was no significant difference between the variants.

The same results were obtained in K562 cells. Indeed, we observed a lower fluorescence level in the *ABCB1* transfected cell lines compared to control cell lines (Fig. 3b, p < 0.001) and these differences were abolished when ABCB1-mediated efflux was inhibited by LY335979 (Fig. 3b, p < 0.001).

### Impact of *ABCB1* 1236C > T-2677G > T-3435C > T polymorphisms on the cytotoxicity of doxorubicin and vinblastine

**Figure 4:**
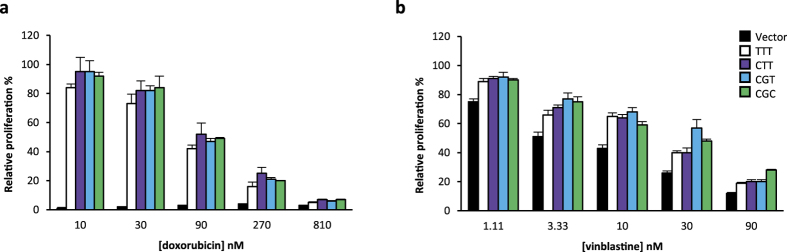
*ABCB1* 1236C > T-2677G > T-3435C > T polymorphisms do not affect the cytotoxicity of doxorubicin and vinblastine. Cell proliferation after treatment with different concentrations of (**a**) doxorubicin or (**b**) vinblastine for 24h (N = 3). The relative proliferation of treated cells compared to control cells is shown.

Since ABCB1 has been reported to transport doxorubicin and vinblastine, we assessed the influence of *ABCB1* variant expression on K562 cell proliferation in the presence of these drugs to further characterize our model. We demonstrated that recombinant cells were more resistant to these anticancer drugs, compared to control cell lines (Fig. 4a [10 to 270 nM], p < 0.001; Fig. 4b, p < 0.001). The effect was particularly spectacular for low doses of doxorubicin. Furthermore, we observed no consistent difference in resistance between K562_C-G-T_, K562_C-T-T_ and K562_T-T-T_ compared to K562_C-G-C_ (Fig. 4, p > 0.05). These observations suggest that variants do not alter the ABCB1 efflux activity towards these two specific substrates.

### Impact of *ABCB1* 1236C > T-2677G > T-3435C > T polymorphisms on anti-proliferative effects of tyrosine kinase inhibitors

**Figure 5:**
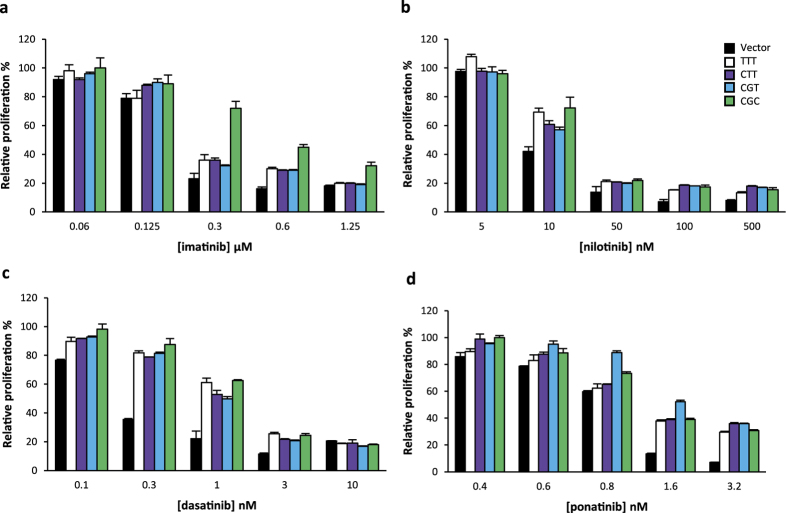
Impact of *ABCB1* 1236C > T-2677G > T-3435C > T polymorphisms on anti-proliferative effects of TKIs. K562 cell proliferation after treatment with different concentrations of (**a**) imatinib, (**b**) nilotinib, (**c**) dasatinib or (**d**) ponatinib for 24h (N ≥ 3). The relative proliferation of treated cells compared to control cells is shown. IC_50_ (nM) are shown in Table 1.

Since ABCB1 has been reported to transport TKIs, we investigated the impact of ABCB1 variant expression on K562 cell proliferation in the presence of TKIs that target BCR-ABL namely imatinib, nilotinib, dasatinib and ponatinib. We observed that recombinant cells were more resistant to imatinib, compared to control cell lines (Fig. 5a), confirming that imatinib is a substrate of ABCB1. We next compared the four *ABCB1* variants. The most striking effect was the resistance of K562_C-G-C_ to imatinib compared with K562_C-G-T_, K562_C-T-T_ and K562_T-T-T_ (Fig. 5a). These results were confirmed by calculating half maximal inhibitory concentration (IC_50_) for each cell line (Table 1). This effect was observed at concentrations that are clinically relevant. Our data suggest an increased activity of the wild-type protein (encoded by the CGC haplotype) towards imatinib compared with variant proteins.

*ABCB1* expression also increased cell proliferation in the presence of nilotinib, dasatinib and ponatinib (Fig. 5). This increase was consistently observed in three independent experiments, although it was statistically significant only for dasatinib (Table 1). K562_C-G-C_, K562_C-G-T_, K562_C-T-T_ and K562_T-T-T_ cells exhibited similar sensitivity to nilotinib (Fig. 5b), dasatinib (Fig. 5c) and ponatinib (Fig. 5d), even though K562_C-G-C_ were slightly more resistant to nilotinib compared to K562_T-T-T_ and other cell lines (IC_50_ of 25.0 *vs* 21.2 nM, respectively; Table 1). These observations suggest that these polymorphisms have a much weaker impact on the transport of nilotinib, dasatinib and ponatinib by ABCB1, compared to imatinib.

### Impact of *ABCB1* 1236C > T-2677G > T-3435C > T polymorphisms on the intracellular accumulation of imatinib and nilotinib

**Figure 6:**
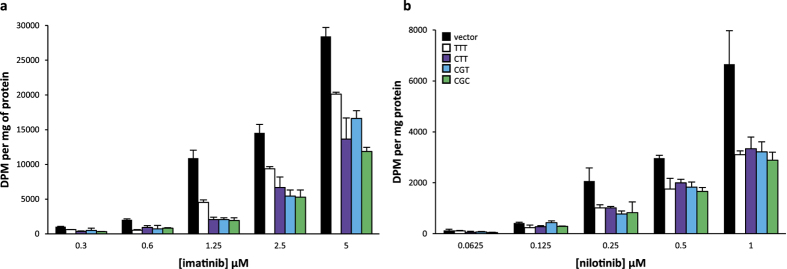
Impact of *ABCB1* 1236C > T-2677G > T-3435C > T polymorphisms on the intracellular accumulation of imatinib and nilotinib. Intracellular accumulation after 120 min of incubation (N = 3) at different concentrations of (**a**) ^14^C-imatinib or (**b**) ^14^C-nilotinib in HEK_pcDNA3.1_, HEK_1236C_ _>_ _T-2677G_ _>_ _T-3435C_ _>_ _T_. The intracellular accumulation of imatinib or nilotinib in each cell line was normalized by reporting the absolute radioactivity on the total amount of proteins in cell extracts (DPM per mg of protein).

We next sought to determine whether these polymorphisms affect the intracellular accumulation of TKIs in transfected cell lines. We obtained radiolabelled imatinib and nilotinib (dasatinib and ponatinb were not available). As depicted in Fig. 6a,b, imatinib or nilotinib intracellular concentrations were strongly decreased in HEK293 transfected cell lines expressing the ABCB1 protein when compared to control cells (Fig. 6a, p < 0.05; Fig. 6b [0.125 to 1 μM], p < 0.05). Expression of the *ABCB1*_T-T-T_ variant haplotype increased accumulation of imatinib when compared to *ABCB1*_C-G-C_ (Fig. 6a [1.25 to 5 μM], p < 0.05), suggesting that this variant affects imatinib efflux. Inconclusive results were obtained with the intermediate haplotypes *ABCB1*_C-G-T_ and *ABCB1*_C-T-T_. Expression of *ABCB1* variants had similar effects on nilotinib accumulation (Fig. 6b, p > 0.05), suggesting that the variants do not alter the transport of nilotinib.

## Discussion

In this study, we show that *ABCB1* 1236C > T-2677G > T-3435C > T polymorphisms affect the sensitivity of human leukemic cells expressing *BCR-ABL* to TKIs, providing a molecular basis for the previously reported associations between this *ABCB1* haplotype and the patient response to imatinib^20^,^21^.

First, HEK293 and K562 recombinant cell lines expressing the *ABCB1*_C-G-C_, *ABCB1*_C-G-T_, *ABCB1*_C-T-T_ or *ABCB1*_T-T-T_ haplotype were generated and carefully validated. As a control, we showed that the four variants decreased Rh123 concentration in a similar manner, in agreement with published data^31^. We also showed that all variants conferred a similar resistance to doxorubicin and vinblastine in concordance to a previous report showing that the efflux of vinblastine is not affected by these *ABCB1* polymorphisms^31^.

We next used these validated models to assess the impact of *ABCB1* variation on TKIs anti-proliferative activity. Our results confirmed that imatinib is a good ABCB1 substrate, as previously described^13^,^32^,^33^,^34^,^35^. Indeed, ABCB1 expression decreased K562 cell sensitivity to imatinib as well as the intracellular accumulation of radiolabelled imatinib in HEK293. Furthermore, we showed that the wild-type protein (ABCB1_C-G-C_) conferred higher imatinib resistance compared to the variant protein (ABCB1_T-T-T_). Consistently, imatinib intracellular concentrations in cells expressing this variant protein were also significantly higher than in cells expressing the wild-type. These observations suggest that the variant haplotype decreases imatinib transport by ABCB1 and provide an explanation for previous *in vivo* studies that associated the wild-type haplotype (CGC) to imatinib resistance^20^,^21^,^22^,^23^.

Nilotinib, dasatinib and ponatinib are also reported as ABCB1 substrates but have been less studied than imatinib in this context^13^,^28^,^29^,^30^,^34^. In our assays, nilotinib intracellular accumulation was much reduced upon ABCB1 expression. The cytotoxicity induced by nilotinib, dasatinib and ponatinib was also decreased, albeit to a variable extent, in cells expressing ABCB1. Moreover, polymorphisms did not affect nilotinib intracellular concentrations and had limited influence on the anti-proliferative effect of these three drugs, in contrast with imatinib. This observation suggests that the studied *ABCB1* polymorphisms significantly affect the cell response to imatinib but not, or to a much lesser extent, to nilotinib, dasatinib, ponatinib, doxorubicin and vinblastine. This was reminiscent of our previous study on *ABCB1* 1199G > A SNP, which had also demonstrated a differential effect of ABCB1 for various substrates^26^.

Among the three investigated coding SNPs, rs1128503 (1236C > T, Gly412Gly), rs2032582 (2677G > T/A, Ala893Ser/Thr), and rs1045642 (3435C > T, Ile1145Ile), only the 2677G > T SNP is associated to an amino acid substitution. Interestingly, in our study, we observed an impact of the 3435C > T synonymous SNP on the anti-proliferative activity of imatinib (see above) but not of the 2677G > T non-synonymous SNP. The functional impact of *ABCB1* 3435C > T synonymous SNP has been clarified in several studies^36^,^37^,^38^. Indeed, despite the fact that this is a synonymous SNP, this variant was shown to alter the kinetics of translation of the protein via the insertion of a rare codon. More precisely, this SNP was suggested to create a translational pause, which could slowdown translation by the ribosome and modify the kinetics of protein folding by chaperones^36^. We could however not confirm the importance of this particular polymorphism in the imatinib intracellular accumulation assay, possibly because this test is less sensitive and more prone to experimental variations.

Although the resistance to TKIs conferred by ABCB1 expression was not as striking as for doxorubicin, adapting imatinib dose according to *ABCB1* genotype might be interesting for CML patients. Moreover, we showed that 2^nd^ generation TKIs are less dependent on the studied *ABCB1* genetic polymorphisms, which are very frequent in the Caucasian population. Future studies should establish whether *ABCB1* genotype affects the inter-patient variability of the response to TKI.

In summary, our *in vitro* results show that the *ABCB1* 1236C > T-2677G > T-3435C > T polymorphisms affect the anti-proliferative activity and the intracellular accumulation of imatinib.

## Material and Methods

### Material

Imatinib, nilotinib and dasatinib were purchased from LC Laboratories (Woburn, United States). Ponatinib was purchased from Selleckchem (Munich, Germany). Radioactive ^14^C-imatinib (specific activity 0.14 mCi/mmol) and ^14^C-nilotinib (specific activity 52 mCi/mmol) were a kind gift from Novartis Pharma (Vilvoorde, Belgium). LY335979 (Zosuquidar 3HCL) was purchased from Bio-connect (Huissen, Netherlands). Rhodamine 123 (Rh123) was obtained from Sigma-Aldrich (St-Louis, United States). Doxorubicin and vinblastine were purchased from Pfizer (Brussels, Belgium) and Teva (Wilrijk, Belgium), respectively.

### Generation of *ABCB1* plasmids

The expression vector pcDNA3.1 containing *ABCB1*_1236T-2677T-3435T_ cDNA (thereafter called *ABCB1*_T-T-T_) was a kind gift from Dr Rodney Ho (University of Washington). The plasmids designated *ABCB1*_C-T-T_, *ABCB1*_C-G-T_ and *ABCB1*_C-G-C_ were generated by site-directed mutagenesis using the QuickChange II XL Site-directed mutagenesis kit (Agilent Technologies). The mutated plasmid designated *ABCB1*_1236C-2677T-3435T_ was generated with the mismatched primers 5′-GTT AAG ATC TTG AAG GGC CTG AAC CTG AAG GTG CA-3′ (forward) and 5′-TGC ACC TTC AGC TTC AGG CCC TTG ATC TTA AC-3′ (reverse). The mutated plasmid designated *ABCB1*_1236C-2677G-3435T_ was generated by the mismatched primers 5′-AAG AAA GAA CTA GAA GGT GCT GGG AAG ATC GCT ACT-3′ (forward) and 5′-CAG TAG CGA TCT TCC CAG CAC CTT CTA GTT CTT TCT T-3′ (reverse). The plasmid designated *ABCB1*_1236C-2677G-3435G_ was generated by the mismatched primers 5′-GTG GTG TCA CAG GAA GAG ATC GTG AGG GCA GC-3′ (forward) and 5′-GCT GCC CTC ACG ATC TCT TCC TGT GAC ACC AC-3′ (reverse).

*ABCB1*_C-G-C_ was then subcloned into the pEF-myc-cyto vector using the Xho1 and Not1 restriction sites and mutagenesis was performed as described above.

The mutated plasmid designated *ABCB1*_1236C-2677G-3435T_ with the mismatched primers 5′-CAG GAA GAG ATT GTG AGG GCA-3′ (forward) and 5′-GCT GCC CTC ACA ATC TCT TCC-3′ (reverse). The mutated plasmid designated *ABCB1*_1236C-2677T-3435T_ was generated by the mismatched primers 5′-AAG AAA GAA CTA GAA GGT TCT GGG AAG ATC GCT ACT G-3′ (forward) and 5′-CAG TAG CGA TCT TCC CAG AAC CTT CTA GTT CTT TCT T-3′ (reverse). The mutated plasmid designated *ABCB1*_1236T-2677T-3435T_ was generated by the mismatched primers 5′-GTT AAG ATC TTG AAG GGT CTG AAC CTG AAG GTG CA-3′ (forward) and 5′-TGC ACC TTC AGC TTC AGA CCC TTG ATC TTA AC-3′ (reverse).

*ABCB1* was fully sequenced after each mutagenesis to confirm the presence of the desired mutation.

### Generation of stable recombinant cell lines

HEK293 and K562 cell lines were obtained from ATCC and grown as previously described^26^. HEK293 and K562 cell lines were transfected with pcDNA3.1 and pEF-myc-cyto vectors, respectively and further selected in the presence of G418 (pcDNA3.1, 1 mg/ml and pEF-myc-cyto, 1.5 mg/ml) according to our previously published method^26^,^39^. Cells were then sorted as described below.

### Characterization of ABCB1 expression

#### Flow cytometry

This experiment was performed as previously described^26^ with minor changes. For each experiment, 5 × 10^5^ cells (HEK293 and K562) were harvested by centrifugation. Cells were washed with ice-cold HAFA solution [filtrated (0.22 μm) Hank’s buffer with 3% decomplemented FBS and NaN_3_ (20 mmol/l)]. Then, cells were resuspended in HAFA solution containing the primary FITC mouse anti-P-glycoprotein antibody diluted 1:10 (clone17F9 557002, BD Pharmingen) or its matched isotypic control diluted 1:10 (FITC mouse IgG 2bk, clone27–35 555742, BD) and incubated 45 min on ice in the dark. Cells were further washed with HAFA solution, centrifuged and finally fixed in 1:1 HAFA/paraformaldehyde (4% in PBS, Affymetrix). Samples were analysed on a Fluorescence-activated cell sorting (FACS) Canto II (BD). Life cell sorting was performed using the same protocol without NaN_3_ and paraformaldehyde.

#### Immunofluorescence

This assay was performed as previously reported^26^, with slight modifications. One day before the experiment, HEK293 cells were plated at a density of 5 × 10^4^ cells/well in complete medium. The next day, cells were washed with PBS/0.1% BSA, fixed with paraformaldehyde 4% during 15 min and rinsed with PBS/0.1% BSA. Cell membranes were subsequently permeabilized with 0.1% Triton X100 during 5 min. After a washing step with PBS/0.1% BSA, cells were incubated with the primary monoclonal antibody Ab4E3 (ab10333, Abcam, 5 μg/ml, diluted with PBS/0.1% BSA) or with its isotopic control (Mouse Ig2a kappa Monoclonal, ab10353, Abcam, 25 μg/ml) for 90 min in the dark. Cells were washed twice and incubated for 60 min with goat anti-mouse IgG coupled to FITC (ab6785, Abcam, 1 μg/ml) and with DAPI (Hoechst 33258 pentahydrate (bis-benzimide), H3569, Invitrogen). Finally, cells were fixed a second time with paraformaldehyde 4% during 5 min. Fluorescence was analysed in fluorescent mounting medium (Dako) with a digital Evos microscope (AMG, Westburg).

### Thymidine incorporation assay

K562 cells were seeded at 10^4^ cells/well (96 well plate) in complete medium and incubated for 24 h at 37 °C with doxorubicin, vinblastine, imatinib, nilotinib, dasatinib or ponatinib at different concentrations. One μCi of ^3^H-thymidine (2 Ci/mmole) was then added to each well and further incubated for 24 h. The radioactivity was measured with a TopCount NXT liquid scintillation counter (PerkinElmer). Proliferation in the presence of drugs was divided by the proliferation of control cells to obtain the relative proliferation rate. To obtain dose-inhibition curve and for determination of IC_50_, we fitted our data into Hill equation:


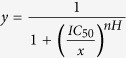


where y is the percentage of inhibition of proliferation compared to untreated cells, IC_50_ is the concentration that is supposed to produce 50% inhibition, x is the drug concentration in logarithm and nH is the Hill coefficient. The coefficients where estimated by nonlinear adjustment with the use of JMP Pro version12 statistical Software based on the maximum likelihood estimation.

### TKI accumulation

One day before the experiment, 3.5 × 10^5^ transfected HEK293 cells were seeded in poly-L-lysine-coated 24-well plates in complete medium. ^14^C-imatinib or ^14^C-nilotinib were diluted with an equal amount of cold compound and added at five different total concentrations (from 0.3 to 5 μM and from 0.0625 to 1 μM, respectively) and cells were incubated for 120 min at 37 °C, 5% of CO_2_. After incubation with imatinib or nilotinib, the cells were washed two times in cold PBS. After centrifugation, the supernatant was discarded and cells were detached with ice-cold lysis buffer (0.1% triton X100 and 0.1% sodium deoxycholate). Radioactive imatinib or nilotinib were quantified in cell lysis using a Tri-Carb liquid scintillation βcounter (Perkin Elmer), after addition of 4 ml of Ultima Gold liquid scintillation cocktail. The absolute amount of drug present in cell extracts was normalized to the amount of protein as quantified using the BCA kit (Thermo Scientific).

### Statistical analysis

Experiment results are presented as mean with standard deviation. GraphPad InStat (Version 3.05) was used for statistical analyses (Fig. 3 and Table 1). Analyses of variance were used under the null hypothesis that the means of the compared groups were equal. Student-Newman-Keuls tests were performed when the differences between means were significant. JMP Pro (Version 11) was used for statistical analyses (Figs 4). Drug concentrations (in DPM per mg protein) and relative cell proliferation (%) of different cell lines were compared with a mixed-model analysis. The model was built on the maximum likelihood ratio, with cell line as the fixed factor, the replicate as the subject analysed and tested drug concentration as the repeated measurement. No particular structure was imposed on the variances and covariances between and within the tested concentrations of the repeated measurements. To test the overall effect of the haplotype, Dunnett post-Hoc comparison was used with empty vector transfected control sets as the reference. When indicated (see results), we restricted the statistical analyses over a range of tested concentrations (nM or μM) for which ABCB1 effect was noticeable and not saturated.

## Additional Information

**How to cite this article**: Dessilly, G. *et al.* Impact of ABCB1 1236C > T-2677G > T-3435C > T polymorphisms on the anti-proliferative activity of imatinib, nilotinib, dasatinib and ponatinib. *Sci. Rep.* **6**, 29559; doi: 10.1038/srep29559 (2016).

## Figures and Tables

**Table 1:** Impact of ABCB1 1236C > T-2677G > T-3435C > T polymorphisms on anti-proliferative effects of TKIs.

ITK	Cell line	IC_50_(nM) ± SEM
Imatinib	vector	257 ± 34
	TTT	459 ± 95*
	CTT	419 ± 70*
	CGT	435 ± 84*
	CGC	732 ± 95^*,§^
Nilotinib	vector	13.7 ± 3.0
	TTT	21.2 ± 3.0
	CTT	18.9 ± 2.2
	CGT	19.5 ± 2,4
	CGC	25.0 ± 2.6*
Dasatinib	vector	0.33 ± 0.05
	TTT	1.31 ± 0.11***
	CTT	1.06 ± 0.04***
	CGT	1.08 ± 0.05***
	CGC	1.43 ± 0.03***^,^^#^
Ponatinib	vector	0.83 ± 0.08
	TTT	1.15 ± 0.26
	CTT	1.18 ± 0.25
	CGT	1.41 ± 0.39
	CGC	1.17 ± 0.22

Experiments were performed as in Fig. 5. Mean IC_50_ were calculated from three independent experiments. *p < 0.05; **p < 0.01; ***p < 0.001 when compared to empty vector. ^§^Significantly different (p < 0.05) from all other *ABCB1* variants; ^#^significantly different from K562_CGT_ and K562_CTT_.

## References

[b1] Medves S. & Demoulin J. B. Tyrosine kinase gene fusions in cancer: translating mechanisms into targeted therapies. Journal of cellular and molecular medicine 16, 237–248, doi: 10.1111/j.1582-4934.2011.01415.x (2012). 21854543 PMC3823288

[b2] Frankfurt O. & Licht J. D. Ponatinib - a step forward in overcoming resistance in chronic myeloid leukemia. Clinical cancer research: an official journal of the American Association for Cancer Research, doi: 10.1158/1078-0432.CCR-13-0258 (2013). 23935038

[b3] Deremer D. L., Ustun C. & Natarajan K. Nilotinib: a second-generation tyrosine kinase inhibitor for the treatment of chronic myelogenous leukemia. Clinical therapeutics 30, 1956–1975, doi: 10.1016/j.clinthera.2008.11.014 (2008). 19108785

[b4] Tothova E. *et al.* Imatinib mesylate in Philadelphia chromosome-positive, chronic-phase myeloid leukemia after failure of interferon alpha. Neoplasma 52, 63–67 (2005). 15739029

[b5] An X. *et al.* BCR-ABL tyrosine kinase inhibitors in the treatment of Philadelphia chromosome positive chronic myeloid leukemia: a review. Leukemia research 34, 1255–1268, doi: 10.1016/j.leukres.2010.04.016 (2010). 20537386

[b6] Goldman J. M. & Melo J. V. Chronic myeloid leukemia–advances in biology and new approaches to treatment. The New England journal of medicine 349, 1451–1464, doi: 10.1056/NEJMra020777 (2003). 14534339

[b7] Stegmeier F., Warmuth M., Sellers W. R. & Dorsch M. Targeted cancer therapies in the twenty-first century: lessons from imatinib. Clinical pharmacology and therapeutics 87, 543–552, doi: 10.1038/clpt.2009.297 (2010). 20237469

[b8] Kantarjian H. M., Talpaz M., Giles F., O’Brien S. & Cortes J. New insights into the pathophysiology of chronic myeloid leukemia and imatinib resistance. Annals of internal medicine 145, 913–923 (2006). 17179059 10.7326/0003-4819-145-12-200612190-00008

[b9] Balabanov S., Braig M. & Brummendorf T. H. Current aspects in resistance against tyrosine kinase inhibitors in chronic myelogenous leukemia. Drug discovery today. Technologies 11, 89–99, doi: 10.1016/j.ddtec.2014.03.003 (2014). 24847658

[b10] Bixby D. & Talpaz M. Mechanisms of resistance to tyrosine kinase inhibitors in chronic myeloid leukemia and recent therapeutic strategies to overcome resistance. Hematology/the Education Program of the American Society of Hematology. American Society of Hematology. Education Program 461–476, doi: 10.1182/asheducation-2009.1.461 (2009). 20008232

[b11] Vasiliou V., Vasiliou K. & Nebert D. W. Human ATP-binding cassette (ABC) transporter family. Human genomics 3, 281–290 (2009). 19403462 10.1186/1479-7364-3-3-281 PMC2752038

[b12] Ueda K., Cardarelli C., Gottesman M. M. & Pastan I. Expression of a full-length cDNA for the human “MDR1” gene confers resistance to colchicine, doxorubicin, and vinblastine. Proceedings of the National Academy of Sciences of the United States of America 84, 3004–3008 (1987). 3472246 10.1073/pnas.84.9.3004 PMC304789

[b13] Mahon F. X. *et al.* MDR1 gene overexpression confers resistance to imatinib mesylate in leukemia cell line models. Blood 101, 2368–2373, doi: 10.1182/blood.V101.6.2368 (2003). 12609962

[b14] Mahon F. X. *et al.* Evidence that resistance to nilotinib may be due to BCR-ABL, Pgp, or Src kinase overexpression. Cancer research 68, 9809–9816, doi: 10.1158/0008-5472.CAN-08-1008 (2008). 19047160

[b15] Gromicho M. *et al.* Development of imatinib and dasatinib resistance: dynamics of expression of drug transporters ABCB1, ABCC1, ABCG2, MVP, and SLC22A1. Leukemia & lymphoma 52, 1980–1990, doi: 10.3109/10428194.2011.584005 (2011). 21663515

[b16] Cascorbi I. *et al.* Frequency of single nucleotide polymorphisms in the P-glycoprotein drug transporter MDR1 gene in white subjects. Clinical pharmacology and therapeutics 69, 169–174, doi: 10.1067/mcp.2001.114164 (2001). 11240981

[b17] Schwab M., Eichelbaum M. & Fromm M. F. Genetic polymorphisms of the human MDR1 drug transporter. Annual review of pharmacology and toxicology 43, 285–307, doi: 10.1146/annurev.pharmtox.43.100901.140233 (2003). 12359865

[b18] Li Y. H., Wang Y. H., Li Y. & Yang L. MDR1 gene polymorphisms and clinical relevance. Yi chuan xue bao = Acta genetica Sinica 33, 93–104, doi: 10.1016/S0379-4172(06)60027-9 (2006). 16529292

[b19] Haufroid V. Genetic polymorphisms of ATP-binding cassette transporters ABCB1 and ABCC2 and their impact on drug disposition. Current drug targets 12, 631–646 (2011). 21039333 10.2174/138945011795378487

[b20] Dulucq S. *et al.* Multidrug resistance gene (MDR1) polymorphisms are associated with major molecular responses to standard-dose imatinib in chronic myeloid leukemia. Blood 112, 2024–2027, doi: 10.1182/blood-2008-03-147744 (2008). 18524988

[b21] Au A. *et al.* Association of genotypes and haplotypes of multi-drug transporter genes ABCB1 and ABCG2 with clinical response to imatinib mesylate in chronic myeloid leukemia patients. Biomedicine & pharmacotherapy = Biomedecine & pharmacotherapie 68, 343–349, doi: 10.1016/j.biopha.2014.01.009 (2014). 24581936

[b22] Zu B. *et al.* MDR1 gene polymorphisms and imatinib response in chronic myeloid leukemia: a meta-analysis. Pharmacogenomics 15, 667–677, doi: 10.2217/pgs.13.222 (2014). 24798723

[b23] Zheng Q. *et al.* ABCB1 polymorphisms predict imatinib response in chronic myeloid leukemia patients: a systematic review and meta-analysis. The pharmacogenomics journal 15, 127–134, doi: 10.1038/tpj.2014.54 (2015). 25245580

[b24] Dickens D., Owen A., Alfirevic A. & Pirmohamed M. ABCB1 single nucleotide polymorphisms (1236C > T, 2677G > T, and 3435C > T) do not affect transport activity of human P-glycoprotein. Pharmacogenetics and genomics 23, 314–323, doi: 10.1097/FPC.0b013e328360d10c (2013). 23619510

[b25] Skoglund K., Moreno S. B., Baytar M., Jonsson J. I. & Green H. ABCB1 haplotypes do not influence transport or efficacy of tyrosine kinase inhibitors *in vitro*. Pharmacogenomics and personalized medicine 6, 63–72, doi: 10.2147/PGPM.S45522 (2013). 24019750 PMC3760445

[b26] Dessilly G. *et al.* ABCB1 1199G > A genetic polymorphism (Rs2229109) influences the intracellular accumulation of tacrolimus in HEK293 and K562 recombinant cell lines. Plos one 9, e91555, doi: 10.1371/journal.pone.0091555 (2014). 24621983 PMC3951418

[b27] Elens L. *et al.* Functional defect caused by the 4544G > A SNP in ABCC2: potential impact for drug cellular disposition. Pharmacogenetics and genomics 21, 884–893, doi: 10.1097/FPC.0b013e32834d672b (2011). 22027652

[b28] Dohse M. *et al.* Comparison of ATP-binding cassette transporter interactions with the tyrosine kinase inhibitors imatinib, nilotinib, and dasatinib. Drug metabolism and disposition: the biological fate of chemicals 38, 1371–1380, doi: 10.1124/dmd.109.031302 (2010). 20423956 PMC2913625

[b29] Hegedus C. *et al.* Interaction of nilotinib, dasatinib and bosutinib with ABCB1 and ABCG2: implications for altered anti-cancer effects and pharmacological properties. British journal of pharmacology 158, 1153–1164, doi: 10.1111/j.1476-5381.2009.00383.x (2009). 19785662 PMC2785536

[b30] Sen R. *et al.* The novel BCR-ABL and FLT3 inhibitor ponatinib is a potent inhibitor of the MDR-associated ATP-binding cassette transporter ABCG2. Molecular cancer therapeutics 11, 2033–2044, doi: 10.1158/1535-7163.MCT-12-0302 (2012). 22778153 PMC3683995

[b31] Fung K. L. *et al.* MDR1 Synonymous Polymorphisms Alter Transporter Specificity and Protein Stability in a Stable Epithelial Monolayer. Cancer research, doi: 10.1158/0008-5472.CAN-13-2064 (2014). PMC4784985 24305879

[b32] Illmer T. *et al.* P-glycoprotein-mediated drug efflux is a resistance mechanism of chronic myelogenous leukemia cells to treatment with imatinib mesylate. Leukemia: official journal of the Leukemia Society of America, Leukemia Research Fund, UK 18, 401–408, doi: 10.1038/sj.leu.2403257 (2004). 14724652

[b33] Shukla S., Sauna Z. E. & Ambudkar S. V. Evidence for the interaction of imatinib at the transport-substrate site(s) of the multidrug-resistance-linked ABC drug transporters ABCB1 (P-glycoprotein) and ABCG2. Leukemia: official journal of the Leukemia Society of America, Leukemia Research Fund, UK 22, 445–447, doi: 10.1038/sj.leu.2404897 (2008). 17690695

[b34] Shukla S., Chen Z. S. & Ambudkar S. V. Tyrosine kinase inhibitors as modulators of ABC transporter-mediated drug resistance. Drug resistance updates: reviews and commentaries in antimicrobial and anticancer chemotherapy 15, 70–80, doi: 10.1016/j.drup.2012.01.005 (2012). 22325423 PMC3348341

[b35] Gurney H. *et al.* Imatinib disposition and ABCB1 (MDR1, P-glycoprotein) genotype. Clinical pharmacology and therapeutics 82, 33–40, doi: 10.1038/sj.clpt.6100201 (2007). 17495881

[b36] Kimchi-Sarfaty C. *et al.* A “silent” polymorphism in the MDR1 gene changes substrate specificity. Science 315, 525–528, doi: 10.1126/science.1135308 (2007). 17185560

[b37] Fung K. L. *et al.* MDR1 synonymous polymorphisms alter transporter specificity and protein stability in a stable epithelial monolayer. Cancer research, doi: 10.1158/0008-5472.CAN-13-2064 (2013). PMC4784985 24305879

[b38] Parmley J. L. & Hurst L. D. How do synonymous mutations affect fitness? BioEssays: news and reviews in molecular, cellular and developmental biology 29, 515–519, doi: 10.1002/bies.20592 (2007). 17508390

[b39] Velghe A. I. *et al.* PDGFRA alterations in cancer: characterization of a gain-of-function V536E transmembrane mutant as well as loss-of-function and passenger mutations. Oncogene 33, 2568–2576, doi: 10.1038/onc.2013.218 (2014). 23752188

